# A Critical Blimp-1-Dependent IL-10 Regulatory Pathway in T Cells Protects From a Lethal Pro-inflammatory Cytokine Storm During Acute Experimental *Trypanosoma brucei* Infection

**DOI:** 10.3389/fimmu.2020.01085

**Published:** 2020-06-04

**Authors:** Carl De Trez, Benoit Stijlemans, Viki Bockstal, Jennifer Cnops, Hannelie Korf, Jacques Van Snick, Guy Caljon, Eric Muraille, Ian R. Humphreys, Louis Boon, Jo A. Van Ginderachter, Stefan Magez

**Affiliations:** ^1^Research Unit of Cellular and Molecular Immunology, Vrije Universiteit Brussel (VUB), Brussels, Belgium; ^2^Myeloid Cell Immunology Laboratory, VIB Centre for Inflammation Research, Brussels, Belgium; ^3^Laboratory of Hepatology, Department of Chronic Diseases, Metabolism and Ageing (CHROMETA), KU Leuven, Leuven, Belgium; ^4^de Duve Institute, Université Catholique de Louvain, Brussels, Belgium; ^5^Ludwig Cancer Research, Brussels Branch, Brussels, Belgium; ^6^Laboratory for Microbiology, Parasitology and Hygiene (LMPH), University of Antwerp, Wilrijk, Belgium; ^7^Unité de Recherche en Biologie des Microorganismes, Laboratoire d'Immunologie et de Microbiologie, Université de Namur, Namur, Belgium; ^8^Laboratoire de Parasitologie, Université Libre de Bruxelles (ULB), Brussels, Belgium; ^9^Division of Infection and Immunity/Systems Immunity University Research Institute, Cardiff University, Cardiff, United Kingdom; ^10^Bioceros, Utrecht, Netherlands; ^11^Ghent University Global, Incheon, South Korea

**Keywords:** *T. brucei*, IL-10, inflammation, T cells, IL-27, Blimp-1

## Abstract

In many infectious diseases, the immune response operates as a double-edged sword. While required for protective immunity, infection-induced inflammation can be detrimental if it is not properly controlled, causing collateral body damage and potentially leading to death. It is in this context that the potent anti-inflammatory cytokine interleukin-10 (IL-10) is required to dampen the pro-inflammatory immune response that hallmarks trypanosomosis. Effective control of this infection requires not just the action of antibodies specific for the parasite's variable surface glycoprotein (VSG) coat antigens, but also a pro-inflammatory immune response mediated mainly by IFNγ, TNF, and NO. However, strict control of inflammation is mandatory, as IL-10-deficient mice succumb from an unrestrained cytokine storm within 10 days of a *Trypanosome brucei* infection. The relevant cellular source of IL-10 and the associated molecular mechanisms implicated in its trypanosomosis associated production are poorly understood. Using an IL-10 reporter mouse strain (Vert-X), we demonstrate here that NK cells, CD8^+^ T cells and CD4^+^ T cells as well as B cells and plasma cells constitute potential cellular sources of IL-10 within the spleen and liver during acute infection. The IL-10 wave follows peak pro-inflammatory cytokine production, which accompanied the control of peak parasitemia. Similar results were observed following conventional experimental needle infection and physiological infections via *T. brucei*-infected tsetse flies. Our results show that conditional T cell-specific ablation of the IL-10 regulating *Prdm1* gene (encoding for the Blimp-1 transcription factor), leads to an uncontrolled trypanosome-induced pro-inflammatory syndrome like the one observed in infected IL-10-deficient mice. This result indicates that the biological role of IL-10-derived from non-T cells, including NK cells, is of minor importance when considering host survival. The cytokine IL-27 that is also considered to be an IL-10 regulator, did not affect IL-10 production during infection. Together, these data suggest that *T. brucei* activates a Blimp-1-dependent IL-10 regulatory pathway in T cells that acts as a critical anti-inflammatory rheostat, mandatory for host survival during the acute phase of parasitemia.

## Introduction

During inflammation, immune regulatory cytokines are mandatory to preserve host integrity by controlling pathogen-induced immune responses (1). IL-10 is a pleiotropic anti-inflammatory cytokine that dampens many inflammatory reactions (2). Hence, IL-10-deficient mice are prone to uncontrolled inflammation-mediated immunopathologies, such as spontaneous colitis potentially leading to tumorigenesis and chronic inflammation-driven auto-immunity (3). During infection, the genetic or pharmacological inhibition of IL-10 usually leads to a better control of infection, but this is often associated with enhanced immuno-pathology and, in some infectious models, death (4, 5). Macrophages and dendritic cells constitute targets of the suppressive function of IL-10, promoting their differentiation toward a suppressive and tolerizing phenotype (6, 7). Recently, many cell types have been shown to secrete IL-10 during infections, namely NK cells, dendritic cells, Th1 cells, CD8^+^ T, regulatory T and B cells, and even non-immune cells, such as hepatocytes and keratinocytes (8–15). Different transcription factors and cytokines have been implicated in the production of IL-10 depending on the cell type. For example, Foxp3, Aryl hydrocarbon Receptor, Blimp1 and IL-27 were shown to modulate IL-10 production by Tregs, NK, and Th1 cells, respectively (12, 16–22). African trypanosomes are extracellular protozoan hemoflagellated parasites transmitted by the bite of infected tsetse flies (genus *Glossina*). These parasites are endemic in Sub-Saharan Africa, causing African trypanosomosis in human (also called sleeping sickness) and Nagana disease in livestock. About 60 million people are at risk and Nagana causes three million cattle deaths every year due to weight loss and anemia. The associated economic loss in livestock production is estimated at 4 billion USD per year (23). Murine models are considered valuable tools to study the interactions between parasites and hosts that contribute to immunopathogenicity. Experimental *T. brucei* infections in mice have shown that clearance of the first parasitemia peak is dependent on an early strong type 1 inflammatory immune response, involving IFNγ, Nitric Oxide (NO) and Tumor Necrosis Factor (TNF) production, which correlates with an early activation of monocytes, the recruitment of splenic neutrophils and the development of anemia (24–28). Yet, the production of IL-10 is essential to dampen this type 1 immune response after parasitemia has been cleared to prevent the development of a hyper-inflammation syndrome and death (6, 29, 30). Despite the importance of IL-10 in *T. brucei* pathogenesis, the *in vivo* cellular source of IL-10 and the associated molecular mechanism(s) implicated in its production remain poorly understood. In this study, we report that increasing levels of IL-10 are being measured in both infected tissue and serum following clearance of the first parasitemia peak. Using IL-10 reporter [Vert-X (31)] mice, we show that NK cells, CD8^+^ T cells and CD4^+^ T cells are important cellular sources of IL-10 within infected liver and spleen tissues around day 6 post infection (p.i.), following the peak of pro-inflammatory cytokine production. Post-parasitemia peak (around day 8–9 p.i.), the cellular source of IL-10 is still similar in the liver, whereas, surprisingly, the main splenic IL-10-producing cells become plasma B cells. These results were first obtained in a conventional experimental infection model in which mice were challenged with *T. brucei* parasites via intraperitoneal needle injection. Subsequently, all results were confirmed following a natural infection via *T. brucei*-bearing tsetse flies. Using T cell conditional Blimp-1 knockout mice, we demonstrate the importance of this transcription factor in dampening trypanosome-mediated inflammation, mainly via the control of T cell activation and IL-10 production, and ultimately host survival.

## Materials and Methods

### Ethics Statement

All experiments complied with the ECPVA guidelines (CETS n° 123) and were approved by the VUB Ethical Committee (Permit Number: 14-220-10 and 14-220-05). Breeding and experimental work with tsetse flies was approved by the Scientific Institute Public Health Department Biosafety and Biotechnology (SBB 219.2007/1410). To minimize mouse suffering and distress during blood sampling, all animals were anesthetized with isoflurane using a UNO—Univentor Anesthesia Unit according to the manufacturer‘s protocol. Mice were monitored daily. Humane endpoints were used during the study, based on weight loss, animals with >25% weight loss were sacrificed using carbon dioxide treatment.

### Parasites, Mice, and Infection

Eight to 12 weeks-old male and female wild type C57BL/6 (WT) mice were purchased from Janvier, France. IL-10^−/−^ (B6.129P2-*Il10*^tm1Cgn^/J), Prdm1^eYFP/+^ (B6.Cg-Tg(Prdm1-EYFP)1Mnz/J), Vert-X (B6(Cg)-*Il10*^*tm*1.1*Karp*^/J), and B cell^−/−^ (B6.129S2-*Ighm*^*tm*1*Cgn*^/J) mice were purchased from Jackson Laboratory, USA. *CD4*^*Cre*/+^ *Prdm1*^*fl*/*fl*^ and littermate control *Prdm1*^*fl*/*fl*^ were kindly provided by A. Scheffold at Charité - Universitätsmedizin Berlin, Berlin, Germany. *CD4*^*Cre*/+^ *IL-10*^*fl*/*fl*^ and littermate control *IL-10*^*fl*/*fl*^ mice were initially established in Cardiff University, Cardiff, United Kingdom (32). All mice were bred and maintained in the animal facility at the Vrije Universiteit Brussel.

Pleomorphic T. brucei AnTat 1.1E parasites were obtained from the Institute for Tropical Medicine, Belgium and stored at −80°C as blood aliquots containing 50% Alsever buffer (Sigma-Aldrich) and 10% glycerol (final V/V). Mice were infected with 5000 clonal AnTat1.1E trypanosomes via intraperitoneal (i.p.) injection in a volume of 200 μL PBS. Tsetse flies were infected at the Institute of Tropical Medicine with T. brucei AnTAR1 parasites and selected for mature salivary gland infections as described previously (33). For each mouse, one individual infected tsetse fly was used to initiate a natural infection by a fly bite.

### Serum and Cell Isolation

Blood from non-infected control and infected mice at different time points of infection was harvested via tail-cut using heparinized capillaries and centrifuged at 8,000 g for 15 min. Serum was harvested and stored at −20°C.

Leukocyte liver cells were purified by perfusing the liver with 10 ml of cold PBS via the inferior vena cava, mechanical disruption of the liver, followed by passing cell suspensions over a 70 μm nylon mesh filter. The cells were washed twice with PBS and centrifuged at 582 g for 7 min at 4°C. After discarding the supernatant, the pellet was resuspended in a 33% Percoll solution and centrifuged at 394 g for 7 min at room temperature. After discarding the supernatant, the pellet was resuspended using ACK lysis buffer (0.15 M NH_4_Cl, 1.0 mM KHCO_3_, 0.1 mM Na_2_-EDTA) to lyse red blood cells (RBCs) and centrifuged at 394 g for 7 min at 4°C. The pellet was resuspended in complete medium buffer [RPMI supplemented with 10% FCS, 2 mM L-glutamine, 100 U/ml penicillin and 100 μg/ml streptomycin (all from Invitrogen Life Technologies)]. Spleen and lymph node cells were isolated as follows. The organs were mechanically disrupted and RBCs were lysed using ACK lysis buffer (0.15 M NH_4_Cl, 1.0 mM KHCO_3_, 0.1 mM Na_2_-EDTA). After washing twice with RPMI at 394 g for 7 min at 4°C, the cell suspensions were resuspended in complete medium and passed through a 70 μm nylon mesh filter.

Spleen, liver and lymph node cell populations from non-infected control and infected mice at different time points of infection were counted and cultured *in vitro* for cytokine ELISA or directly analyzed by flow cytometry.

### Flow Cytometry

Cells were centrifuged at 394 g for 7 min and resuspended in FACS medium (5% FCS in PBS) at a concentration of 2.10^6^ cells/ml. Non-specific binding sites were blocked by incubating 20 min. at 4°C with an Fc-blocking antibody (anti-CD16/32, clone 2.4G2). Next, cell suspensions were stained with fluorescent conjugated antibodies for 30 min at 4°C. Fluorescent antibodies: CD11a Pe-Cy7 clone 2D7, CD11b PE-Cy7 clone M1/70, Ly6C APC clone AL-21, CD4 BV421 clone GK1.5, CD8 BV510 clone 53–67, NK11 PE clone PK136, TCRß APC clone H57-597, CD90.2 APC-Cy7 clone Thy1.2, CD138 APC clone 281-2 (BD Biosciences), B220 BV510 clone RA3-6B2, CD93 BV421 clone AA4.1, CD1d PE clone 1B1 and CD49d PE clone 9C10 (eBioscience). Following washing with FACS buffer the cell suspensions were analyzed on a FACS Canto II flow cytometer (BD Biosciences) and data was processed using FlowJo software (Tree Star Inc., Ashland, OR). The total number of live 7-AAD- cells in each population was determined by multiplying the percentages of subsets within a series of marker negative or positive gates by the total live cell number determined by microscopy counting with trypan blue for each tissue.

### Intracellular Cytokine Staining

Spleen and liver cells were incubated in complete medium at 37°C for 4 h in the presence of eBioscience™ Cell Stimulation Cocktail (plus protein transport inhibitors) (500X) (ThermoFisher Scientific). Next, cells were washed in PBS at 394 g for 7 min at 4°C, stained for cell surface markers at 4°C, washed in PBS at 394 g for 7 min at 4°C and fixed in 1x BD Cytofix/Cytoperm solution (BD Pharmingen) for 15–20 min at 4°C. They were then washed using a saponin-based buffer (10 × Perm/Wash in FACS buffer; BD Pharmingen) at 394 g for 7 min at 4°C. After discarding the supernatant, cells were resuspended and stained with XMG1.2 (anti-IFNγ; BD Biosciences) in 1x BD Perm/wash solution for 30 min at 4°C. After washing the cells with 1x BD Perm/wash solution at 1,400 rpm for 7 min at 4°C, they were resuspended in PBS and analyzed on a FACS Canto II flow cytometer (BD Biosciences) and data was processed using FlowJo software (Tree Star Inc., Ashland, OR). No signal was detectable with the allophycocyanin-coupled anti-IgG1 isotype control (eBioscience).

### *In vitro* Cultures

Liver, splenic and lymph node cell populations isolated from infected and uninfected mice resuspended in complete medium buffer (cfr previous “Serum and cell isolation” section) were plated in 48-well plates at 2.10^6^ cells per ml and incubated at 37°C in a 5% CO_2_ incubator for 48 h without any additional stimulation before supernatant was recovered for cytokine ELISA.

### *In vivo* Cell Depletion and Neutralization Experiments

For depletion of CD8^+^ and CD4^+^ T cells, mice received the first i.p. injection of 500 μg anti-CD8 beta and anti-CD4 rat-anti-mouse monoclonal antibodies (clone YTS169 and GK1.5, Bioceros in the Netherlands, respectively) 24 h prior to infection. Subsequently, mice received a dose of 100 μg at 2 days interval post infection (34). NK and NKT cells were depleted with the anti-NK1.1 PK136 mouse-anti-mouse monoclonal antibody (PK136, BioXCell, USA and Bioceros, The Netherlands). Two hundred and fifty microgram was given 4 and 1 day prior to infection. A dose of 300 μg was given at 2–3 day intervals post infection (34). Control mice were treated with a respective control antibody isotype(all from BioXCell, USA), namely a monoclonal rat IgG2a isotype as control for the anti-NK1.1 depletion and a monoclonal mouse IgG2b as control for anti-CD8 and anti-CD4, and the same regimen. Depletion efficiency of NK and NKT cells as well as CD8^+^ and CD4^+^ T cells from both spleen and liver was assessed by flow cytometry and was confirmed to be above 90%.

For neutralization of IL-27, wild type mice were treated with 500 μg of an anti-IL-27 antibody (MM27.7B1) or a control IgG2a antibody once a week (32, 35–37).

### Quantification of Cytokines

Cytokines were quantified using a V-PLEX Custom Mouse Cytokine kit (catalog number K152A0H) from Meso Scale Discovery (Rockville, MD, USA) according to the manufacturer's protocol. Alternatively, culture medium and serum concentrations of IL-6, TNF, IFN-γ, and IL-10 (R&D Systems) were determined by ELISA as recommended by the suppliers.

### Bone Marrow Chimera

Eight weeks-old C57BL/6 mice were irradiated at 1000 Rads using a Cesium source irradiator at IBMM, Gosselies, Belgium. The next day irradiated mice were transplanted intravenously with 10^7^ bone marrow cells isolated from femurs and tibias of either IL-10-deficient mice or wild-type (WT) C57BL/6 mice. Briefly, bones were harvested and cells were flushed with PBS using a 27 gauge needle and syringe. Cell clumps were dissociated via up-and-down pipetting, filtered through a 70 μm nylon mesh filter and washed in PBS at 394 g for 7 min at 4°C. Red blood cells were lyzed using RBC lysis buffer, washed in PBS at 394 g for 7 min at 4°C and counted. Animals were kept under Sulfatrim antibiotic [sulfamethoxazole/trimethoprim, to be added to the drinking water (5/200 ml)] for 4 weekspost-irradiation.

### Statistics

The GraphPad Prism 7 software was used for statistical analyses (student *t*-test for paired analyses and Log-rank (Mantel-Cox) test for survival). Values are expressed as mean ± SEM, where ^*^p ≤ 0.05, ^**^p ≤ 0.01, ^***^p ≤ 0.001, and ^****^p ≤ 0.0001.

## Results

### Clearance of Parasitemia in *T. brucei*-Infected Mice Is Followed by the Production of IL-10 in Spleen and Liver

**Figure 1:**
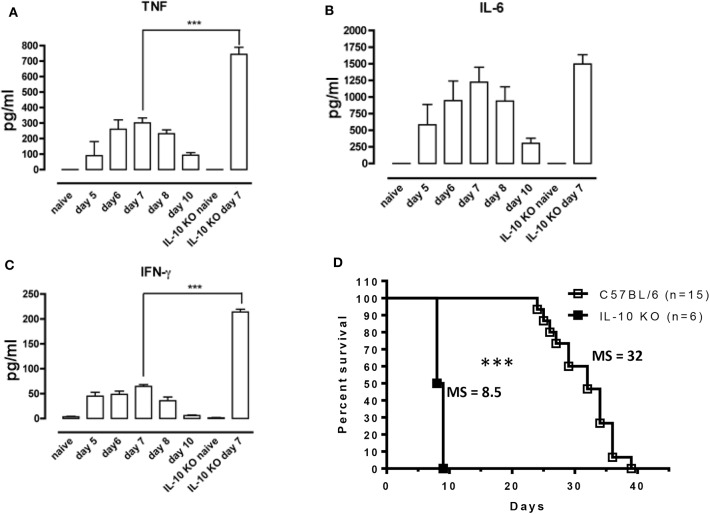
Uncontrolled inflammation in *T. brucei*-infected IL-10-deficient mice. **(A–C)** Cytokine levels were measured by ELISA in plasma of naive mice, infected WT mice on days 5, 6, 7, 8, and 10 p.i. and naive vs. infected IL-10-deficient mice on day 7 p.i. Data are represented as mean of 6 mice per group ± SEM and are representative of 2 independent experiments. **(D)** Survival of WT and IL-10-deficient mice following *T. brucei* infection i.p., where the median survival (MS) of each group is indicated in days. Data are presented as a pool of two representative independent experiments with a minimum of three mice per group, where ***p ≤ 0.001.

In Antat1.1E *T. brucei*-infected wild-type (WT) C57BL/6 mice, peak parasitemia is reached around day 6 post-infection (p.i.) (Supplementary Figure 1) and blood parasitemia control correlates with the development of a pro-inflammatory response characterized by increased levels of TNF, IFNγ, and IL-6, which peaks around day 7 and then decreases toward 10 p.i. (Figures 1A–C). Early after infection, IL-10 production is absolutely necessary to dampen the pathogenic effects of these pro-inflammatory cytokines, as mice deficient in IL-10 (IL-10 KO mice) exhibit increased levels of the pro-inflammatory cytokines IFNγ and TNF (Figures 1A–C), and die around 8–9 days p.i. (Figure 1D). Interestingly, the increased inflammation observed in IL-10 KO mice has no effect on parasitemia levels (6, 29).

**Figure 2:**
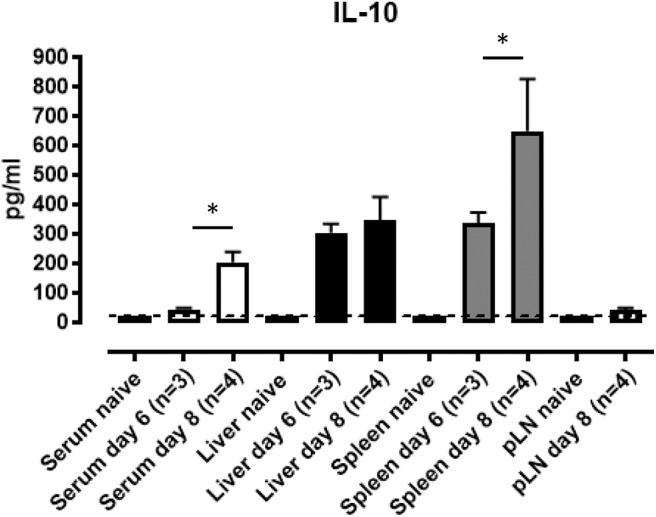
IL-10 production in mouse spleen and liver cell cultures following *T. brucei* infection. Serum and supernatants from *ex vivo* cultured total spleen cells, purified leukocyte liver cells from naïve, day 6 and day 8 p.i and pooled peripheral lymph node (pLN) cells from naïve and day 8 p.i. were analyzed by ELISA for IL-10 production. Data are represented as mean of at least three mice per group ± SEM, where *p ≤ 0.05, and are representative of three independent experiments. The dashed line represents the detection limit.

Given the essential role for IL-10 in controlling *T. brucei* pathogenesis, serum IL-10 levels and IL-10 secretion in *ex vivo* splenic, liver and pooled peripheral lymph node (axillary and inguinal) cell cultures were measured. IL-10 protein in the serum of *T. brucei* infected WT mice peaks around day 8 p.i., which corresponds to the time at which IL-10 KO mice start to succumb to the infection (Figure 2). In spleen and liver cell cultures, local production of IL-10 is detected at day 6 and 8 post-infection without the addition of *ex vivo* stimuli (Figure 2). This contrasts with the nearly complete absence of IL-10 secretion measured in pooled peripheral lymph nodes at the same time points (Figure 2). Together, these results suggest that IL-10 produced in liver and spleen is crucial in dampening pro-inflammatory cytokines induced during parasitemia.

### Identification of IL-10 Producing Cells During Early *T. brucei* Infection

**Figure 3:**
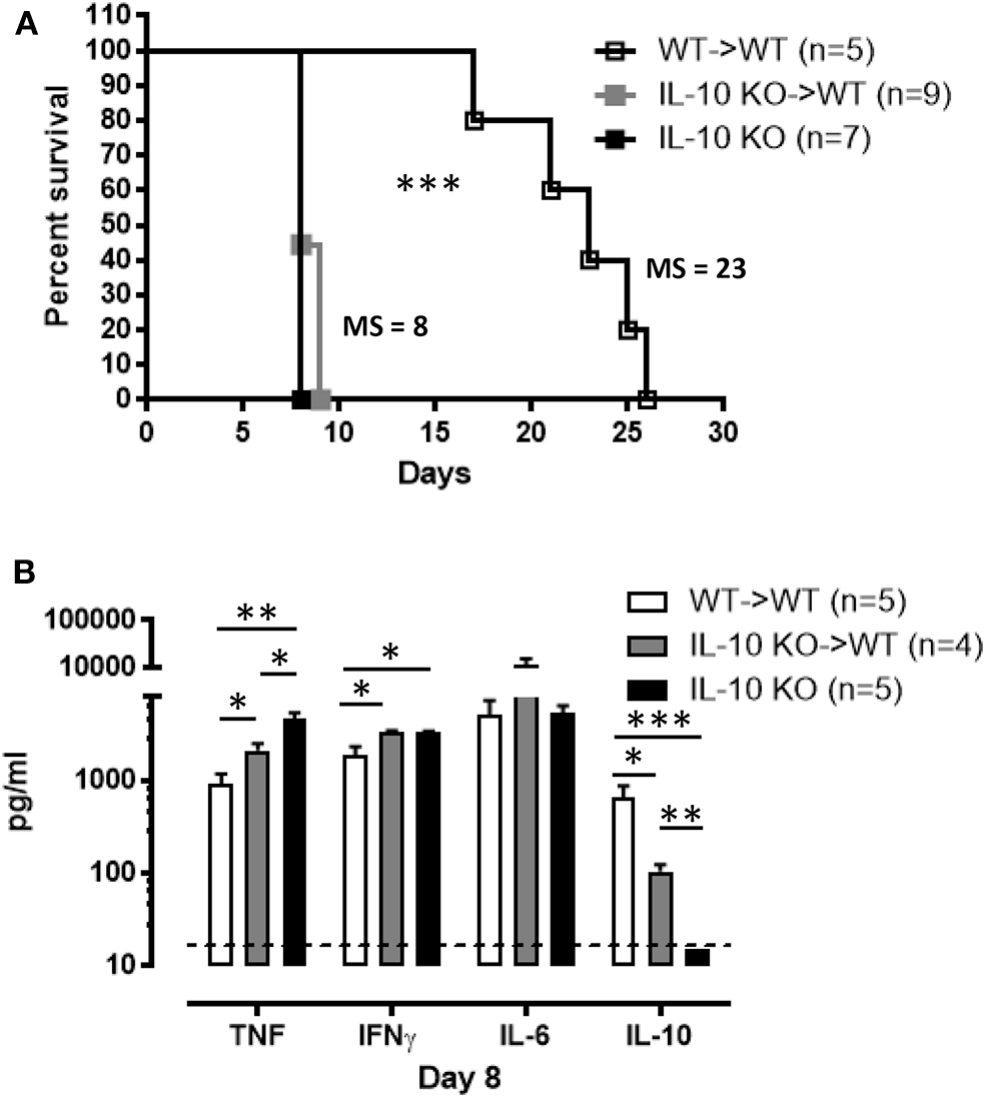
Hematopoietic cells constitute a major source of IL-10 production during *T. brucei* infection. **(A)** Survival of the chimeric mice 8 weeks post-transplantation as well as IL-10-deficient mice following *T. brucei* infection i.p., where the median survival (MS) of each group is indicated in days. **(B)** Cytokine levels were measured by ELISA in plasma of infected chimeric and IL-10-deficient mice 8 days p.i. Data are represented as mean of at least 4 mice per group ± SEM, where *p ≤ 0.05, **p ≤ 0.01, ***p ≤ 0.001, and are representative of 2 independent experiments. The dashed line represents the detection limit.

Numerous cell types, particularly hematopoietic cells, have the potential to produce IL-10. Hence, in order to understand the role of IL-10 in trypanosomosis, the main cellular origin needs to be known. Irradiated WT mice repopulated with bone marrow cells from IL-10 KO mice exhibit comparable susceptibility to *T. brucei*-induced death as compared to full IL-10 KO mice, while a transfer with WT-derived cells completely rescued the phenotype (Figure 3A). The analysis of cytokine levels present in serum at day 8 p.i. in these mice reveals that recipients of IL-10 KO bone marrow display significantly higher IFNγ (3,374 ± 66 pg/ml) and TNF (2,101 ± 83 pg/ml) as compared to WT mice that received WT bone marrow cells (Figure 3B). Coincidingly, a dramatic reduction in infection-associated IL-10 (105 ± 18 pg/ml) is observed (Figure 3B), phenocopying the results observed in IL-10 KO mice. Importantly, the small amount of IL-10 still present in irradiated WT mice receiving IL-10 KO bone marrow cells is insufficient to reduce the overall pro-inflammatory cytokine production in these mice.

**Figure 4:**
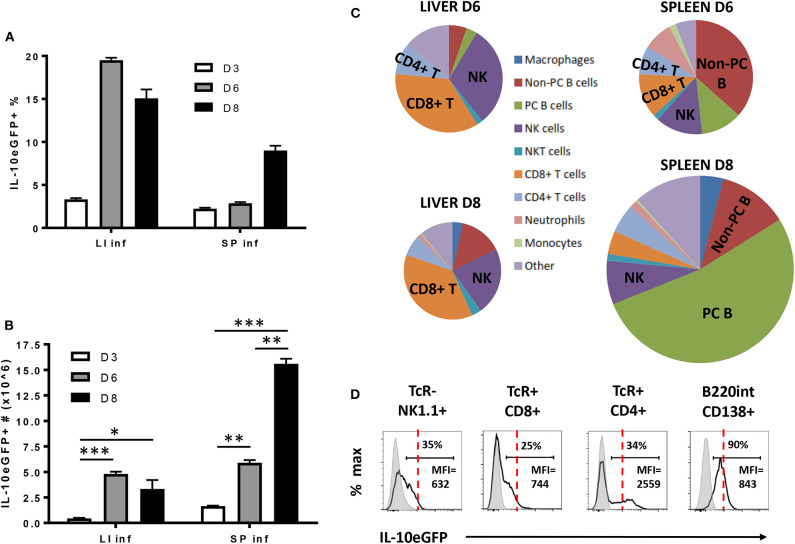
Cellular characterization of IL-10 producing cells in spleen and liver following *T. brucei* infection. **(A)** Percentages and **(B)** total cell number of IL-10 producing cells in infected liver (LI inf) and spleen (SP inf) at day 3, 6, and 8 p.i. **(C)** Relative contribution of the different cellular sources producing IL-10. The surface of the diagram is representative of the total number of IL-10^+^ cells present in liver and spleen at day 6 and day 8 p.i., which is described in **(B)**. **(D)** Histogram showing the intensity of IL-10-eGFP signal as well as the percentage and MFI of IL-10^+^ cells in different cell subsets. The red dotted line is giving an indication of the relative IL-10-eGFP signal differences between the different cell subsets, whereas the gray histogram represent the relative IL-10-eGFP signal of these different cell subsets in infected non-reporter mice. Data are represented as mean of at least three mice per group ± SEM, where *p ≤ 0.05, **p ≤ 0.01, ***p ≤ 0.001, and are representative of 3 independent experiments.

As IL-10 derived from the hematopoietic compartment is mandatory to counteract the excessive production of pro-inflammatory cytokines during early *T. brucei* infection and prevent early death, the radiosensitive source(s) of IL-10 was investigated using VERT-X IL-10eGFP reporter mice, encoding enhanced GFP (eGFP) in the 3' UTR of the *Il10* gene (31). The specificity of the IL-10eGFP signal was tested in both liver and spleen, as infected non-reporter WT mice did not show any increase in eGFP signal compared to Vert-X IL-10eGFP reporter mice (Supplementary Figure 2). First, we follow the evolution of the IL-10eGFP signal expressed in percentage and total numbers within both the liver and spleen during the first 8 days p.i. (Figures 4A,B). In the liver a slight increase in the percentage of IL-10eGFP^+^ cells was observed toward day 3 p.i., peaking at day 6 p.i. and declining slightly around day 8 p.i. In the spleen, the percentage of IL-10eGFP^+^ cells only increase considerably at day 8 p.i. (Figure 4A). In absolute numbers of IL-10eGFP^+^ cells, there is a similar increase in both liver (4.8 10^6^ ± 0.2) and spleen (5.9 10^6^ ± 0.3) at day 6 p.i. compared to day 3 p.i. In the case of the spleen, but not the liver, we observed a substantial further increase in IL-10eGFP^+^ cell numbers by day 8 after infection (Figure 4B). These results mirror the IL-10 production data recorded in the corresponding *ex vivo* cultures of these organs (Figure 2).

Investigating the cellular source of the IL-10eGFP signal at day 6 and day 8 p.i. in both liver and spleen shows that NK cells (NK1.1^+^ TcRαβ^−^) and CD8^+^ T (CD8α^+^ TcRαβ^+^) cells are the main source of IL-10 at day 6 p.i. (30.7% ± 0.3% and 35.2% ± 9.1% of all eGFP^+^ cells, respectively), as well as at day 8 p.i. (22.4% ± 2.3% and 37.0% ± 1.8%, respectively) in livers of infected mice (Figure 4C). Interestingly, NK cells (NK1.1^+^ TcRαβ^−^) and CD8^+^ T (CD8α^+^ TcRαβ^+^) cells were also both recently identified as the main producers of IFNγ in response to *T. brucei* infection (34), In the spleen, the main IL-10eGFP+ cell populations are non-plasma B cells (B220^hi^ CD138^−^) at day 6 p.i. (36.9% ± 4.7%), whereas IL-10^+^ plasma cells (B220^int^ CD138^+^) predominant (52.9% ± 4.5%) at day 8 p.i. with nearly all plasma B cells showing positive IL-10eGFP expression at the later timepoint (Figure 4D). Importantly, CD4^+^ T cells show a double expression profile, with the IL-10 positive population expressing a 3- to-4-fold increased mean fluorescence intensity (MFI) compared to the other subsets (red dashed line) (Figure 4D), suggesting a higher IL-10 expression by this cell subset.

These data reveal that different cell types, such as NK cells, CD8^+^ T cells, and CD4^+^ T cells, and B cells, have the potential to produce IL-10 during early experimental trypanosomosis.

### Natural Tsetse Fly-Mediated *T. brucei* Infection Induces the Same IL-10 Producing Cells as the Intraperitoneal Infection

**Figure 5:**
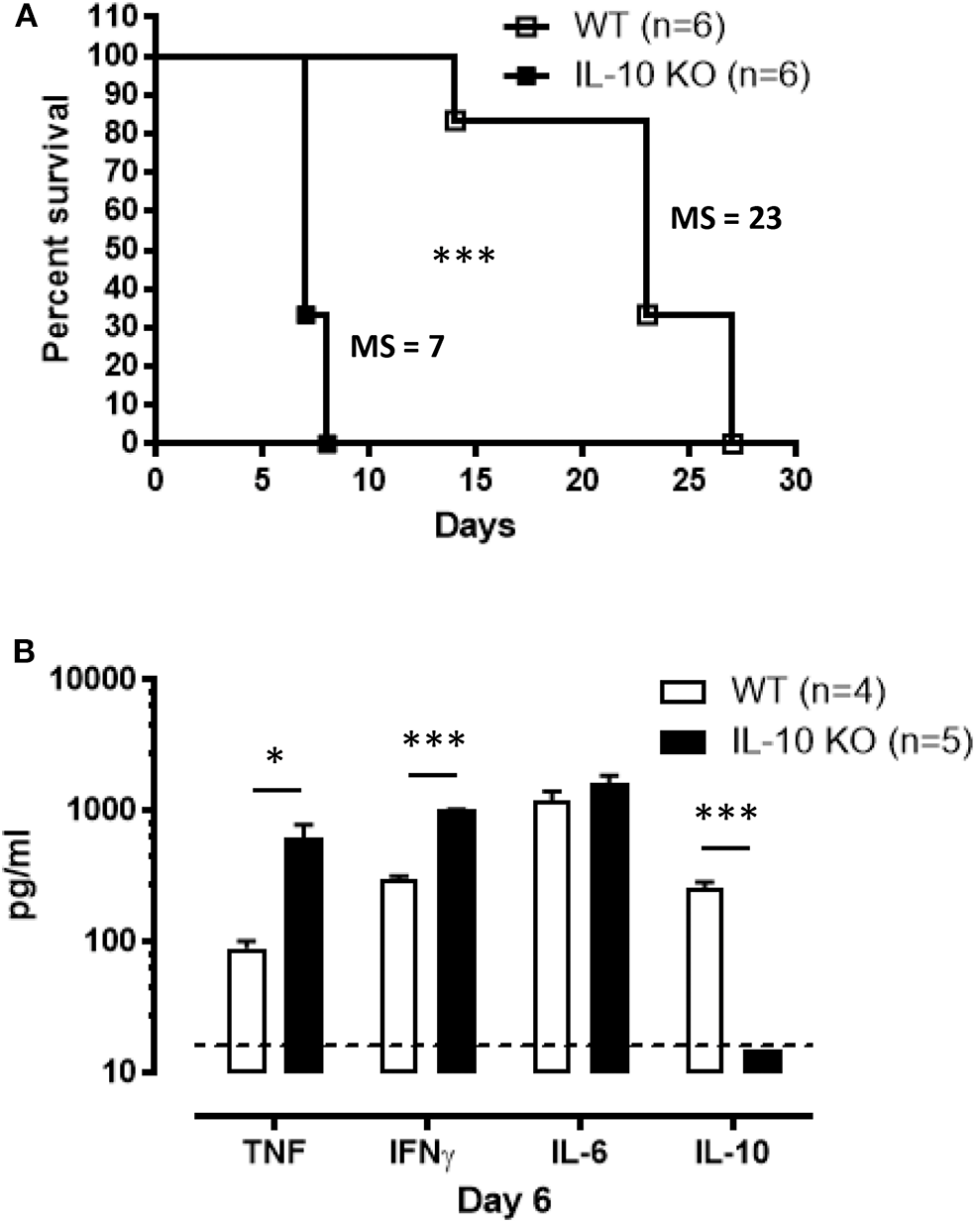
Survival and inflammation profile of IL-10-deficient mice inoculated with *T. brucei* infected tsetse fly. **(A)** Survival of WT and IL-10-deficient mice after *T. brucei* infection via tsetse fly, where the median survival (MS) of each group is indicated in days. Data are represented as mean of at least 6 mice per group ± SEM and are representative of 2 independent experiments. **(B)** Cytokine levels were measured by ELISA in plasma of infected WT and IL-10-deficient mice at 6 days p.i. Data are represented as mean of at least four mice per group ± SEM, where *p ≤ 0.05, ***p ≤ 0.001, and are representative of three independent experiments. The dashed line represents the detection limit.

In most experimental trypanosomosis studies, mice are infected by intraperitoneal (i.p.) injections of parasites. To evaluate the relevance of our results under a more biologically relevant condition, the survival of IL-10-deficient mice following *T. brucei*-infected tsetse fly-mediated infection was monitored. IL-10 KO mice infected via the natural route all succumb within 10 days p.i., similar to intraperitoneally infected IL-10 KO mice (Figure 5A). In agreement with the results obtained following i.p. infection (Figure 1), tsetse fly-mediated infection induces a significant increase in IFNγ and TNF levels, which is further aggravated in IL-10 KO mice, at day 6 p.i. (Figure 5B).

**Figure 6:**
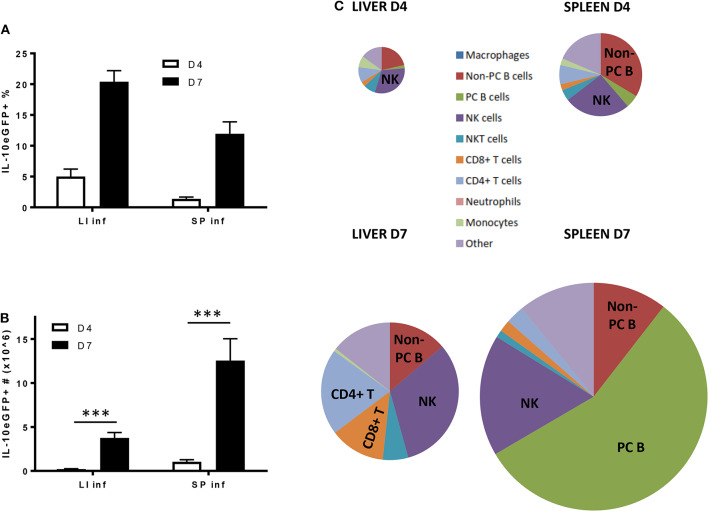
Cellular characterization of IL-10 producing cells in spleen and liver following *T. brucei* infection by tsetse fly. **(A)** Percentages and **(B)** total cell number of IL-10 producing cells in infected liver (LI inf) and spleen (SP inf) at day 4 and 7 p.i. **(C)** Relative contribution of the different cellular sources producing IL-10. The surface of the diagram is representative of the total number of IL-10^+^ cells present in liver and spleen at day 4 and day 7 p.i., which is described in **(B)**. Data are represented as mean of at least three mice per group ± SEM, where ***p ≤ 0.001, and are representative of three independent experiments.

Following the natural route of a *T. brucei* infection, the liver exhibits the highest frequency of IL-10eGFP^+^ cells, as compared to the spleen, both at day 4 and day 7 p.i. (Figure 6A). However, due to the occurrence of splenomegaly, the spleen contains a higher actual number of IL-10eGFP^+^ cells at day 7 p.i. as compared to the liver (12.6 10^6^ ± 2.5 vs. 3.8 10^6^ ± 0.6, respectively) (Figure 6B), which mirrors the results obtained following i.p. infection. Also here, NK cells, CD8^+^ T cells, CD4^+^ T cells subsequently followed by non-plasma and plasma B cells remain the most abundant cell types producing IL-10 at day 7 p.i., the time point when IL-10 KO mice infected via the natural route start to succumb from the pro-inflammatory cytokine storm (Figure 6C).

### Relative Importance of the IL-10-Producing Cell Subsets Following *T. brucei* Infection

**Figure 7:**
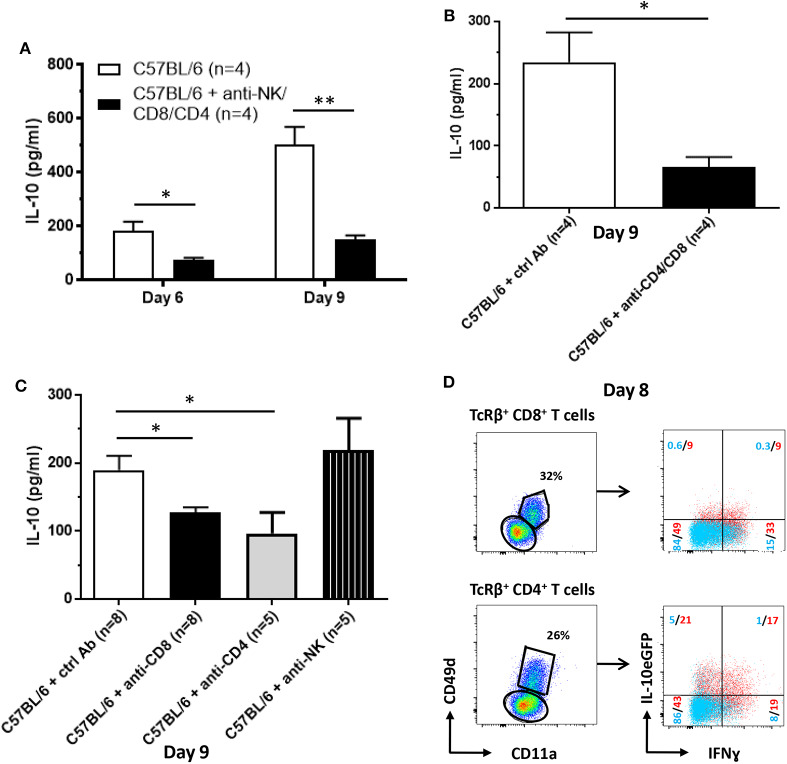
Cellular source of IL-10 production following *T. brucei* infection. IL-10 levels were measured by ELISA in plasma of infected WT and anti-NK-, anti-CD8- and CD4-treated WT at day 6 and 9 p.i. **(A)** as well as in WT and anti-CD8- or CD4-treated or anti-NK1.1-treated WT mice **(B)** and in WT and anti-CD8- and CD4-treated 9 days p.i. **(C)**. **(D)** At day 8 p.i., CD11a and CD49d expression on splenic CD8^+^ and CD4^+^ T cells was analyzed. Both CD11a^+^ CD49d^+^ and CD11a^−^ CD49d^−^ CD8^+^ and CD4^+^ T cell subsets were analyzed for the expression of IL-10eGFP and intracellular expression of IFNg. Data are represented as mean of at least four mice per group ± SEM, where *p ≤ 0.05, **p ≤ 0.01, and are representative of two independent experiments.

As shown by the data compiled above, NK and T cells constitute a potential early source of IL-10 during *T. brucei* infection (Figure 4). Therefore, these cells were pharmacologically depleted and subsequently the levels of IL-10 in the circulation at day 6 and day 9 post-infection were measured (Figure 7A). The results confirm the contribution of NK and T cells on systemic IL-10 levels at both day 6 (184 ± 34 pg/ml vs. 76 ± 7 pg/ml) and day 9 (502 ± 68 pg/ml vs. 152 ± 15 pg/ml) post-infection. As we confirmed that the peak of IL-10 in the serum occurs post peak parasitemia, round day 8–9 post infection, the role of these individual cell subsets, i.e., NK cells as well as CD8 and CD4 T cells, on the circulating levels of IL-10 was evaluated at day 9 post-infection (new Figure 7B). The results demonstrate that both CD8^+^ and CD4^+^ T cells, in contrast to NK cells, play a role in the systemic production of IL-10 at day 9 post-infection.

Taken the importance of both T cell subsets and the very high level of IL-10eGFP expression in the cytokine positive CD4^+^ T cell population, the role of these cells was assessed in more detail using *T. brucei* infected mice, which are pharmacologically depleted in both CD8^+^ and CD4^+^ T cells. In these mice, serum IL-10 levels at day 9 p.i.were 70–75% lower compared to WT mice (234 ± 48 pg/ml vs. 66 ± 16 pg/ml) (Figure 7C). Together, these results confirm that T cells constitute an important source of systemic IL-10 as well as the data obtained at day 9 p.i. in Figure 7A.

To further characterize these IL-10-producing T cells, they were assessed for their levels of CD11a and CD49d expression. These are typical markers for activated and antigen-experienced effector type 1 regulatory T (Tr1) cells that are known for their combined capacity to produce both IFNγ and IL-10 (38–40). Figure 7D shows that ~18 and 38% of the antigen-experienced CD11a^+^ CD49d^+^ CD8^+^ and CD4^+^ T cells (red), respectively, are IL-10eGFP^+^, whereas <1 and 6% of the non-antigen experienced CD8^+^ and CD4^+^ T cells (blue) are IL-10eGFP^+^ at day 8 p.i. (Figure 7D). Moreover, within the IL-10eGFP^+^ T cell populations, 50% of the CD8^+^ T cells and 45% of the CD4^+^ T cells co-express IFNγ, implying the presence of Tr1 cells in *T. brucei*-infected WT mice.

### IL-10 Production Following *T. brucei* Infection Occurs Independently of IL-27

**Figure 8:**
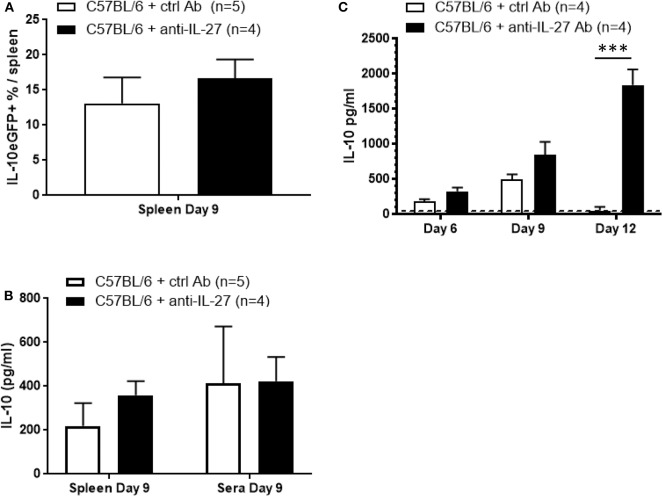
IL-27-independent IL-10 production following *T. brucei* infection. **(A)** Splenic percentage of IL-10eGFP^+^ as well as **(B)** IL-10 levels in supernatant of *ex vivo* spleen cell cultures and in serum were assessed in untreated and anti-IL-27-treated infected Vert-X mice. IL-10 levels were measured by ELISA in plasma of infected WT and anti-IL-27-treated WT at day 6, 9, and 12 p.i. **(C)**. Data are represented as mean of minimum 4 mice per group ± SEM, where ***p ≤ 0.001, and are representative of at least TWO independent experiments.

To gain further insight into the mechanism of IL-10 upregulation following *T. brucei* infection the role of IL-27 was assessed. This member of the IL-12 cytokine family has been shown to promote IL-10 production by various T cell subsets via IL-27 receptor engagement and subsequent STAT3-dependent signaling (19, 41, 42). However, blocking IL-27, using an anti-IL-27 neutralizing antibody, in *T. brucei*-infected Vert-X mice did not alter frequencies of splenic IL-10eGFP^+^ cells, nor did it change the IL-10 levels in *ex vivo* cultured spleen cells or in serum at day 9 p.i. (Figures 8A,B). Hence measurement of systemic IL-10 production either before (day 6) or after (day 12) the day 9 peak shows a gradual increase of IL-10 during the course of the anti-IL-27 treatment and *T. brucei* infection (Figure 8C), which is in line with previous results (43). Also, similar to published data obtained in the *T. congolense* model using IL-27R-deficient mice (43), blocking of IL-27 during *T. brucei* infection results in an increased pro-inflammatory cytokine storm characterized by increased serum levels of IFNγ (886 ± 52 pg/ml vs. 156 ± 17 pg/ml) and TNF (353 ± 18 pg/ml vs. 202 ± 5 pg/ml), which is associated with a shortened survival (15 days vs. 55 days in control antibody-treated mice) (Supplementary Figures 3A,B). Together, these data does confirm the importance of IL-27 with respect to its anti-inflammatory role during *T. brucei* infection. However, in contrast to the expectation, the data show that IL-27 does not provide a co-stimulatory signal for the actual infection-associated IL-10 production by T cells or the induction of IL-10eGFP^+^ cells.

### Blimp-1 Expression in T Cells Is Required to Regulate Inflammation Following *T. brucei* Infection

Recently, numerous reports have demonstrated a role for the transcriptional regulator B lymphocyte-induced maturation protein-1 (Blimp-1), encoded by the *Prdm1* gene, in T cell homeostasis and function, but also in the control of systemic inflammation predominantly via the regulation of IL-10 production (17, 18, 44, 45). However, Blimp-1 has also been described to directly dampen T cell and NK cell activation and proliferation as well as the production of pro-inflammatory cytokines such TNF and IFNγ (45–47). In addition, plasma cells are known to express high levels of Blimp-1, as this factor is mandatory for the differentiation of B cells into antibody secreting cells (48, 49). Using Blimp-1eYFP reporter mice (Blimp-1^eYFP/+^), we found that the major cellular sources of Blimp-1 are similar to those producing IL-10eGFP during the first 9 days of infection, namely NK cells and T cells in the liver at day 6 p.i. as well as mainly T cells in the liver and plasma B cells in the spleen, at day 9 p.i (Supplementary Figure 4).

**Figure 9:**
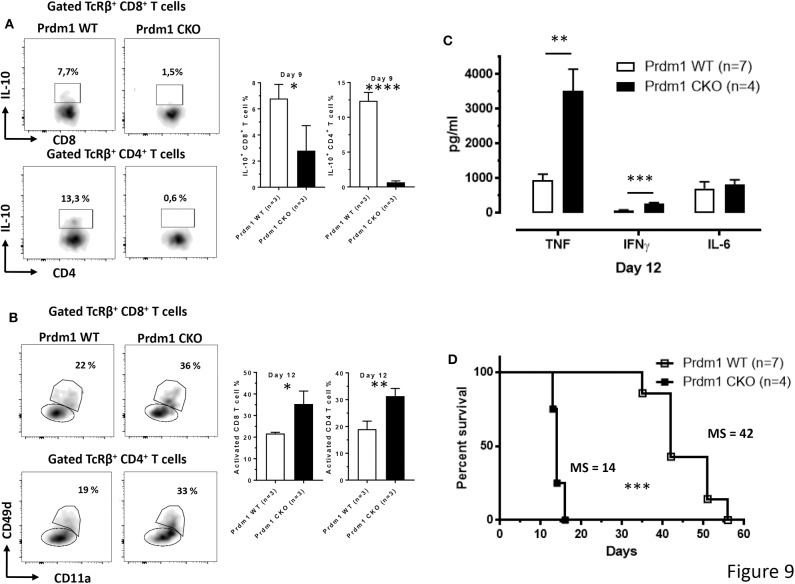
T cell-derived Blimp-1 signaling pathway is required to dampen inflammation following *T. brucei* infection. **(A)** Percentage of splenic IL-10^+^ CD8^+^ and CD4^+^ T cell subsets was analyzed at day 9 p.i. both in WT and T cell-specific Prdm1-deficient (Prdm1 CKO) mice following *T. brucei* infection. **(B)** The CD11a and CD49d expression on splenic CD8^+^ and CD4^+^ T cells were analyzed at day 12 p.i. in WT and T cell-specific Prdm1-deficient (Prdm1 CKO) mice. Data are represented as mean of at least 3 mice per group ± SEM and are representative of 3 independent experiments. **(C)** Cytokine levels in serum at day 12 p.i. and **(D)** survival of WT and Prdm1 CKO mice were monitored following *T. brucei* infection, where the median survival (MS) of each group is indicated in days. Data are represented as mean of at least four mice per group ± SEM, where *p ≤ 0.05, **p ≤ 0.01, ***p ≤ 0.001, ****p ≤ 0.0001, and are representative of two independent experiments.

To evaluate a possible correlation between IL-10 production and the expression of the *Prdm1* gene in T cells, the production of IL-10 by both CD8^+^ and CD4^+^ T cells was analyzed in mice with a conditional deletion of *Prdm1* within the T cell lineage (*CD4*^*Cre*/+^ *Prdm1*^*fl*/*fl*^, Prdm1 CKO) and littermate *CD4*^+/+^ *Prdm1*^*fl*/*fl*^ controls (Prdm1 WT) following *T. brucei* infection. Interestingly, the loss of *Prdm1* expression in T cells leads to an almost complete absence of IL-10 production by splenic CD8^+^ and CD4^+^ T cell subsets compared to WT mice at day 9 p.i. (Figure 9A). As mentioned previously, *Prmd1* is also implicated in dampening T cell activation and pro-inflammatory cytokine production. This is confirmed by the finding that during *T. brucei* infection of Prdm1 CKO mice, an increased frequency of activated antigen-experienced splenic CD8^+^ and CD4^+^ T cells is observed by day 12 p.i. (based on CD11a and CD49d expression) (Figure 9B). Consequently, these mice also display increased serum level of pro-inflammatory cytokines, e.g., IFNγ (205 ± 26 pg/ml vs. 79 ± 19 pg/ml) and TNF (3518 ± 625 pg/ml vs. 1,040 ± 188 pg/ml), at day 12 p.i. (Figure 9C). This sustained systemic increase in pro-inflammatory cytokines is associated with a premature death of infected conditional KO mice (Figure 9D).

## Discussion

The crucial role of IL-10 in dampening pro-inflammatory responses in order to avoid immunopathology is well-established (4, 50). In the case of *T. brucei* infection, IL-10 is necessary to down-modulate the inflammatory response and to prevent death of the host from a hyper-inflammation syndrome. Indeed, IL-10 KO mice started to succumb on day 8 p.i. due to the uncontrolled presence of high pro-inflammatory cytokine levels, such as IFNγ and TNF. In contrast, the IL-6 cytokine does not seem to play a major role in this phenomenon as we did not detect any significant difference in IL-6 levels between IL-10 KO and WT mice in nearly all experimental settings. In some infectious settings, the absence of IL-10 can be associated to the development of immunopathology mediated by the pro-inflammatory IL-17 cytokine, which is mainly driven by the IL-6 production. For example, the absence of IL-10 leads to IL-17-mediated immunopathology during infection with the intracellular *Leishmania major* parasite (51). However, our unpublished observation shows that the lack of IL-10 does not induce any systemic IL-17 levels above detection limit, which correlates with similar IL-6 levels observed between IL-10 KO and WT mice during *T. brucei* infection.

Until now, the cell types responsible for IL-10 secretion in the *T. brucei* trypanosome infection model have never been well-characterized. Since many immune hematopoietic-derived cells have the capacity to produce IL-10 (8–10, 14, 52), we first confirmed that the radiosensitive bone marrow compartment is the main source of this cytokine. Also, day 8 p.i. coincides with the highest level of IL-10 in the serum and *ex vivo* liver and spleen cell cultures, whereas no IL-10 could be detected in cell cultures from peripheral and mesenteric lymph nodes. Moreover, the data obtained on IL-10 production from *ex vivo* cell cultures correlates with the total number of IL-10eGFP+ cells in liver and spleen at day 6 and 8 p.i. using the Vert-X mice. For example, no difference in the IL-10 level and IL-10eGFP^+^ cell number were observed at day 6 p.i. in liver and spleen as well as day 8 p.i. in liver.

In order to characterize the different immune cell types producing IL-10 in these tissues at different time points after infection, Vert-X IL-10eGFP reporter mice were used. The study identified NK, T and B cells as producers of IL-10 during the acute phase of *T. brucei* infection. For example, in the liver, the main sources of IL-10 are NK and CD8^+^ T cells, both at early and later time points. The early production of IL-10 by NK cells also plays a crucial role during the infection with rapidly disseminating parasites, e.g., *Toxoplasma gondii, Listeria monocytogenes*, and *Yersinia pestis* (8) as well as during the late stage of murine visceral leishmaniasis (53), by counteracting the production of inflammatory cytokines such as IL-12. Another study also reported the anti-inflammatory IL-10 property of antiviral effector CD8^+^ T cells in the lung during acute influenza virus infection (13). In the spleen, non-plasma and plasma B cells predominate as a source of IL-10 at day 6 and 8 p.i., respectively. Recently, many B cell types, such as T2-MZP cells (CD19^+^CD21^hi^CD23^hi^CD1d^hi^), B10 cells (CD5^+^CD1d^hi^), and MZB cells (CD23^−^CD21^hi^CD1d^hi^), were shown to possess intrinsic regulatory properties mainly via the production of IL-10 (54–56). Madan et al. already described that CD19^+^ B cells represent a dominant population of IL-10-expressing cells in the spleen after diverse *in vivo* stimuli, such as lipopolysaccharide, CpG, goat anti-IgD and mouse cytomegalovirus infection (31). IL-10-producing plasmablasts can also exert a regulatory function in autoimmune inflammation (57). However, the protective role of B cell-derived IL-10 was negligible in our experimental model, at least during the acute phase of infection, as the genetic deletion of B cells does not significantly impact the levels of IL-10 in the serum at day 8 p.i., a time point when IL-10 KO mice starts to succumb from an uncontrolled inflammatory response.

Our results also demonstrated that IL-10 KO mice infected with *T. brucei* via the natural route, using tsetse flies, succumbed with a similar kinetic from an uncontrolled pro-inflammatory cytokine storm, mainly consisting of IFNγ and TNF. In addition, the different IL-10-producing cell types in this model are comparable to the ones following i.p. administration of *T. brucei*. Together, these results fit with the hypothesis that systemically disseminating parasites induce a chronological cell programming, leading to IL-10 production in order to counterbalance the production of pro-inflammatory cytokines and to avoid premature host death.

### Cellular and Molecular Regulation of IL-10 Production

The resistance to *T. brucei* infection relies on the development of a typical Type 1 immune response characterized by the production of pro-inflammatory cytokines such as IFNγ and TNF (24, 58). However, this inflammatory response, which is mainly driven by effector T cells, must be tightly regulated in order to avoid immunopathology and potentially an early death. Our study demonstrates that NK and T cells constitute sequential sources of IL-10. These are the exact same cell types that have recently been shown to produce IFNγ following *T. brucei* infection (34). Effector NK and T cells possess the capacity to produce both inflammatory and anti-inflammatory cytokines in various pathogenic models. For example, Tr1 cells can produce IL-10 and IFNγ at the same time (10, 18, 20, 59). In our murine model, we have shown that antigen-experienced IFNγ^+^ CD11a^+^ CD49d^+^ T cells are the main producers of IL-10, suggesting that this activated CD4^+^ T cell subset corresponds to Tr1 cells. The genetic and pharmacological depletion of T cells drastically reduced the levels of IL-10 in the serum of *T. brucei*-infected mice.

As IL-27 is known to play an important role in the regulation of IFNγ during trypanosome infection as well as to modulate IL-10 production in other models (19, 32, 41, 42), we investigated whether IL-27 was affecting IL-10 levels during *T brucei* infection. As previously shown by the group of Shi in the *T. congolense* model (43), we did not find any clear role for IL-27 in IL-10 production during the early stages of *T. brucei* infection. For example, within the first 12 days p.i. during African trypanosomosis, the levels of IL-10 in serum and *ex vivo* spleen culture supernatant from infected anti-IL-27-treated mice were similar, or even higher at day 12 p.i., to the ones monitored in untreated WT mice. In addition, the percentage of IL-10+ cells is also similar between both groups. Therefore, the exact immunoregulatory role of IL-27 in the context of African trypanosomosis remains ambiguous. However, different studies focusing on the anti-inflammatory role of IL-27 in the context of parasitic infections, such as malaria, *Trypanosoma cruzi* and African Trypanosomosis, but also in viral infection, assign an important role of IL-27 in the regulation of CD4^+^ T cell activation and recruitment, to avoid IFNγ-mediated immunopathology (43, 60–63). For example, during cytomegalovirus infection, IL-27 signaling restricts the development of virus-specific CD4^+^ T cells displaying a cytotoxic phenotype via the inhibition of the transcription factor T-bet expression (63).

Overall, these data suggest that T cells play an important role in the generation of an early IL-10-mediated anti-inflammatory response, which occurs independently of IL-27.

*Prdm1*, the gene coding for Blimp-1, is a master regulator of plasma cell differentiation (49, 64) and, more recently, the expression of Blimp-1 in CD8^+^ and CD4^+^ T cells has been demonstrated to regulate homeostasis and activation via induction of IL-10 and dampening of IL-2 and IFNγ expression (17, 45). Using Blimp-1eYFP reporter mice, we demonstrated that Blimp-1-expressing cells largely overlap with IL-10 expressing cells, namely NK cells and T cells in liver and plasma B cells in spleen. Importantly, *T brucei*-infected mice harboring a conditional deletion of *Prdm1* in T cells succumbed from uncontrolled inflammation within 2 weeks p.i. Numerous recent publications have identified Blimp-1 as a major regulator of T cell activation, mainly by controlling IL-10 and pro-inflammatory cytokine production by both CD8^+^ and Tr1 CD4^+^ T cells (18, 20, 44, 46, 47). In our *T. brucei* infection model, we demonstrated a similar role for *Prdm1* in positively regulating IL-10 expression in T cells as well as dampening their activation and subsequent pro-inflammatory status.

Together, these results suggest that the production of IL-10 during acute *T. brucei* infection constitutes a tightly regulated process both at the cellular and molecular level. Our data demonstrates that the production of IL-10 by CD8^+^ and CD4^+^ T cells is required to dampen the production of pro-inflammatory cytokines by these same cell types early after infection. This study also highlights the importance of the *Prdm1* transcription factor within the T cell compartment, which controls their IL-10 production, their activation status as well as the production of inflammatory cytokines. In conclusion, *Prdm1* acts independently of IL-27 as a master regulator of inflammation during *T. brucei* infection.

## Data Availability Statement

All datasets generated for this study are included in the article/Supplementary Material.

## Ethics Statement

All experiments complied with the ECPVA guidelines (CETS n° 123) and were approved by the VUB Ethical Committee (Permit Number: 14-220-10 and 14-220-05).

## Author Contributions

CD, BS, VB, JC, HK, and GC performed the research work. CD, VB, and JC analyzed the data. JVS, IH, LB, and EM provided materials. CD, BS, JVS, EM, IH, LB, JVG, and SM participated in writing of the manuscript.

## Conflict of Interest

LB was employed by company Bioceros. The remaining authors declare that the research was conducted in the absence of any commercial or financial relationships that could be construed as a potential conflict of interest.
