# Supplementary material for: A Critical Blimp-1-Dependent IL-10 Regulatory Pathway in T Cells Protects From a Lethal Pro-inflammatory Cytokine Storm During Acute Experimental Trypanosoma brucei Infection
Source: Front Immunol. 2020 Jun 4;11:1085. doi: 10.3389/fimmu.2020.01085 (PMC7325990; doi:10.3389/fimmu.2020.01085)
Supplement: Supplementary file 1 [file Data_Sheet_1.PDF]

Supplementary Material

Supplemental Figures

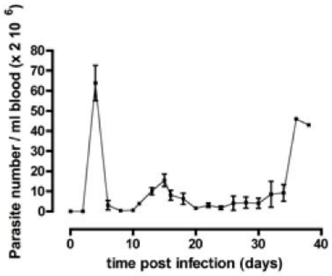

Supplementary Figure 1. Parasitemia in *T. brucei*-infected mice.
Mice were infected by i.p. injection of 5000 *T. brucei* parasites and blood parasitemia was monitored microscopically.

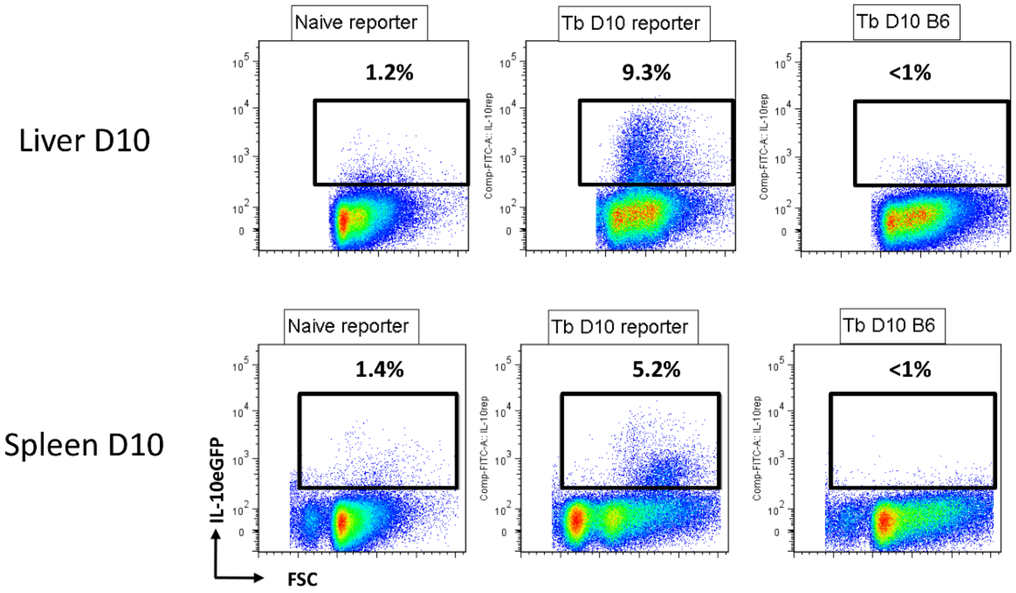

Supplemental Figure 2. IL-10 expression in naive and infected Vert-X and C57BL/6 mice. Naive IL-10 reporter Vert-X mice as well as day 10 *T. brucei*-infected Vert-X and C57BL/6 mice were analyzed for GFP expression in liver and spleen. FACS plots are gated on single live cells.

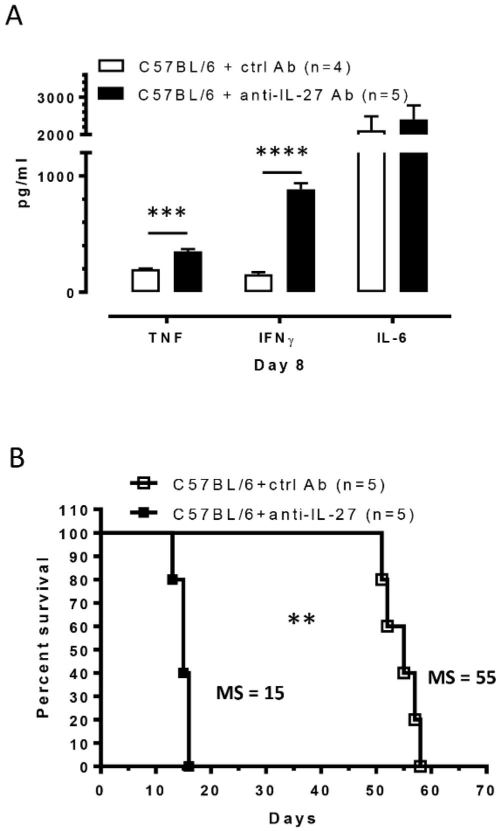

Supplemental Figure 3. IL-27 is required to regulate inflammation and survival following *T. brucei* infection.

(A) Cytokine levels were measured by ELISA in plasma and (B) survival (MS = mean survival) of untreated and anti-IL-27-treated WT mice was monitored following *T. brucei* infection. Data are represented as mean of minimum 4 mice per group ± SEM and are representative of at least 2 independent experiments.

A

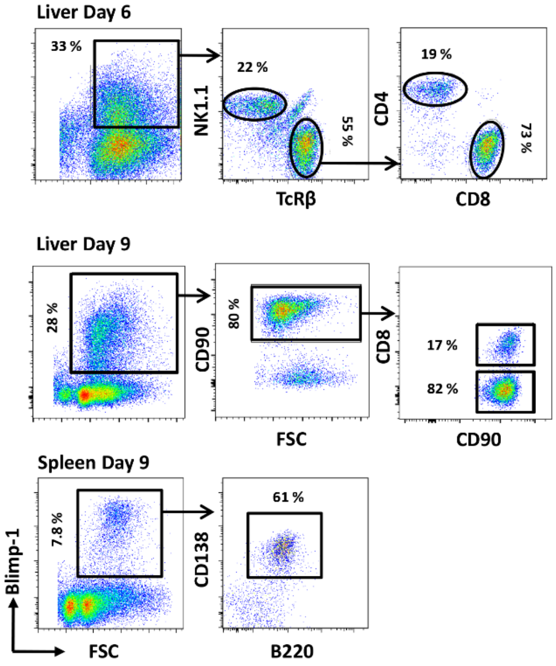

Supplemental Figure 4. Characterization of Blimp-1 expressing cells.
(A) Representative cellular characterization of Blimp-1-expressing cells at day 6 and 9 in infected liver and day 9 in infected spleen based on NK1.1, TcRβ, CD4 and CD8, CD90 and CD8 as well as B220 and CD138, respectively. Data are representative of minimum 3 independent mice per group and of at least 2 independent experiments.
